# The Effects of Cooling and Shrinkage on the Life of Polymer 3D Printed Injection Moulds

**DOI:** 10.3390/polym14030520

**Published:** 2022-01-27

**Authors:** Anurag Bagalkot, Dirk Pons, Digby Symons, Don Clucas

**Affiliations:** Department of Mechanical Engineering, University of Canterbury, Private Bag 4800, Christchurch 8020, New Zealand; digby.symons@canterbury.ac.nz (D.S.); don.clucas@canterbury.ac.nz (D.C.)

**Keywords:** rapid tooling, injection moulding, ejection failure, cooling time, additive manufacturing, tool failure

## Abstract

3D Printed Injection Moulds (3DIM), commonly used for low volume production and prototyping purposes, are known to fail abruptly and have a comparatively shorter life than conventional moulds. Investigating the underlying critical factors affecting failure may help in reducing the risk of abrupt failures and possibly prolong the 3DIM tool life. A hypothesis that the cooling time of the Injection Moulding (IM) process is a critical factor for 3DIM tool failure has been proposed. The failure hypothesis has been validated by theoretical calculations, FEA simulations and experimental investigations. Experiments were performed using two different materials for the 3DIM tool (Visijet M3-X and Digital ABS) and an engineering thermoplastic (Lexan 943-A) as the moulding material. Results showed that cooling time was a critical factor on tool life and managing the thermal loading on a 3DIM tool could lead to increased tool life. The paper identifies cooling time as the critical factor affecting 3DIM tool life and presents a cooling regime that could possibly lead to prolonged tool life.

## 1. Introduction

Injection Moulding (IM) is a polymer processing technique commonly used to mass manufacture parts with complicated geometries for a wide range of industries, 35% by weight of all polymers are processed by IM [1]. Mould tooling is a critical component of the IM process, complex parts require complex tooling which leads to higher costs and longer lead times via conventional manufacturing [2]. For an injection moulded part, the processing and material cost account for a small fraction of the overall part cost, the high upfront tooling represents a major cost [1]. Due to high tooling costs and long lead times, IM was previously considered suitable only for high volume (>10,000 parts) applications. Industries amortised the high upfront tooling costs over the life cycle of the mould. However, for low volume production, the high upfront cost cannot be justified, and industries turn to other manufacturing methods. Traditional moulds are machined out of blocks of steel or aluminium [3]. Based on production quantity, the complexity of the part and budget the common types of moulds used are production moulds (steel) used for high volume (>100,000 parts), insert moulds (aluminium) used for medium volume (10,000–100,000 parts), and rapid moulds (polymer-based 3D printing) used for low volume (10–1000 parts) [3,4]. To reduce the high upfront cost of tooling, the IM industry developed Master Unit Die (MUD) bases [5]. A MUD base is a complex assembly that incorporates a standard frame, ejector plates, ejector pins, core pull mechanisms etc. and pre-machined pockets for mould inserts [6]. A MUD base negates the need for expensive mould base assemblies and instead uses swappable core and cavity inserts. To reduce lead times and cost, industries are experimenting with the use of polymer-based Rapid Tooling (RT) processes to quickly produce mould inserts at relatively cheaper costs compared to traditionally machined mould inserts [7,8,9]. These mould inserts are used in a MUD base, thereby reducing the high upfront tooling costs and lead times making them suitable for prototyping and low volume manufacturing purposes [10].

### 1.1. Rapid Tooling

The use of Additive Manufacturing (AM) technologies to quickly produce tools for prototyping and low volume manufacturing is referred to as Rapid Tooling (RT) [7]. AM technologies utilise a layer-by-layer building process which greatly reduces the wastage of raw material, use of direct labour and complex machining operations thereby reducing both the cost and lead time [11]. Recent improvements in Additive Manufacturing (AM) technologies have led to increased use of RT techniques in the IM industry to create mould tools (core and cavity inserts) for prototyping and low volume manufacturing purposes [12,13]. These mould inserts are also commonly referred to as 3D Printed Injection Moulds (3DIM). RT techniques involve the use of both metal and polymer-based AM systems. The costs of metal AM systems, consumables and raw materials are significantly higher than those for polymer-based AM. Hence most industries utilise polymer-based AM systems for producing 3DIM inserts for prototyping and low volume manufacturing. The SLA-1, developed in 1988 by 3D Systems^®^, used liquid resins (photopolymers) which crosslinked (cured) when exposed to UV light. These were among the first systems that were able to create non-porous parts. Currently, Stereolithography (SLA) and Material Jetting (MJ) are the commonly used AM techniques to produce 3DIM inserts [14]. Other polymer AM systems such as Fused Filament Fabrication (FFF) and Selective Laser Sintering (SLS) have also been experimented with but did not perform well in producing 3DIM inserts due to their porous nature [15,16,17]. SLA techniques have been used for RT since the early 1990s, photopolymers such as Renshape 7510 and Somos 7510 used by early SLA machines had a low heat deflection temperature (*HDT*) of 85 °C [18,19]. The low *HDT* meant that these tools could only be used to mould polymers which required a mould temperature of lower than 85 °C. In addition, the comparatively inferior material properties and high cost of these RT materials compared to traditional tooling materials led to the RT process not garnering widespread industrial use [17].

However, recent advances in the AM industry have led to improvements in speed, printing resolution, build quality and range of available materials [20,21]. These improvements, along with a reduction in the cost of AM equipment over the years, has led to renewed interest in RT.

### 1.2. Failure of 3D Printed Injection Moulds (3DIMs)

Early studies indicated that the properties of the polymer 3DIM materials were not well suited to the demanding thermal conditions of injection moulding applications which lead to failure via thermal degradation [17,19]. The 3DIM failures were usually observed after several moulding cycles, while the process conditions remained the same. It was, therefore suggested that 3DIM failure was not abrupt, but a progressive deterioration leading to failure [17]. The pressure exerted by the incoming polymer flow was also reported to cause deformation and abrupt failures of raised features of 3DIMs [22]. The yield strength of the tool was also reported to be significantly reduced during the moulding process which led to failure [23]. The surface roughness of the 3DIM tools due to the layered manufacturing process was also reported as a significant factor leading to tool failure [24]. Surface erosion was also observed after repeated moulding cycles which lead to excessive flashing and safety issues [25]. Surface smoothening had a positive effect and lead to reduced ejection forces. Coating the 3DIM surface with nickel has been reported to decrease thermal degradation and improve mould life [26]. Infiltration of metals into the 3DIM resins was also carried out to improve the strength of 3DIM tools but was not successful in prolonging the tool life [27].

Although the failure of 3DIMs has been examined from the perspective of mechanical, thermal and material properties, little literature exists on studying the effect of processing conditions on the 3DIM tool life. Recently processing conditions was reported as a significant factor for 3DIM tool life [28,29]. In this paper, we investigate the effect of processing conditions and specifically the effect of the cooling stage on the life of 3DIM tools.

### 1.3. Overview of the Paper

Section 2 of this paper describes the analytical and experimental methodology. Section 3 presents the analytical and experimental results. Section 4 is a discussion of these results. Conclusions with implications for practising engineers on the use of optimum process settings for possibly increasing 3DIM tool life are provided to end the paper.

## 2. Materials and Methods

**Figure 1:**
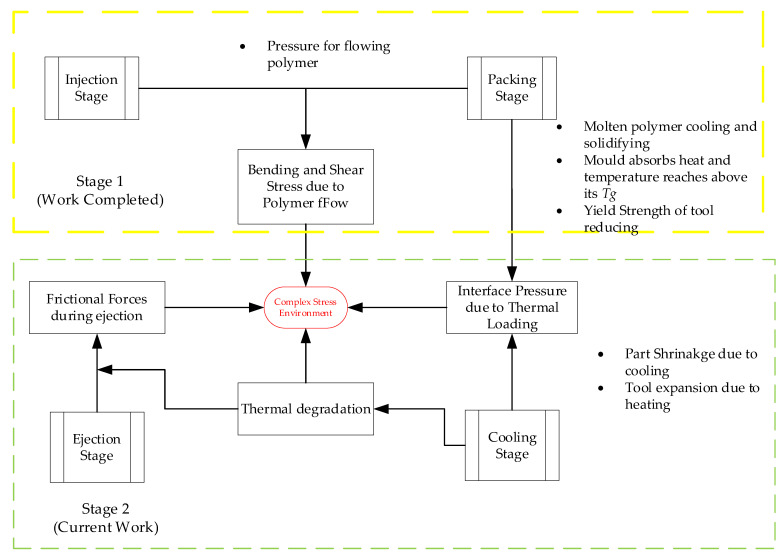
Complex stress environment on 3DIM during different injection moulding stages.

In our previous work, we disproved the common hypothesis from the literature that failure of raised features on 3DIM tools is predominantly due to bending and shear stresses developed due to injection pressure experienced by the tool features [29]. Refer to the yellow block (stage 1) in Figure 1. After further experimentation and examination of failed parts, we propose a new failure hypothesis for raised feature failures of 3DIM. Refer to the green block (stage 2) in Figure 1.

### 2.1. Hypothesised Failure Mode: Pin Failure Due to Interface Pressure and Frictional Forces

**Hypothesis** **1.** *The cooling of polymer (shrinkage) and heating of mould (expansion) during the cooling stage leads to the development of interface pressure. This interface pressure results in higher frictional resistance thereby requiring higher forces to eject the part during the ejection stage. The frictional forces developed during part ejection leads to tensile failure of raised features.*

**Figure 2:**
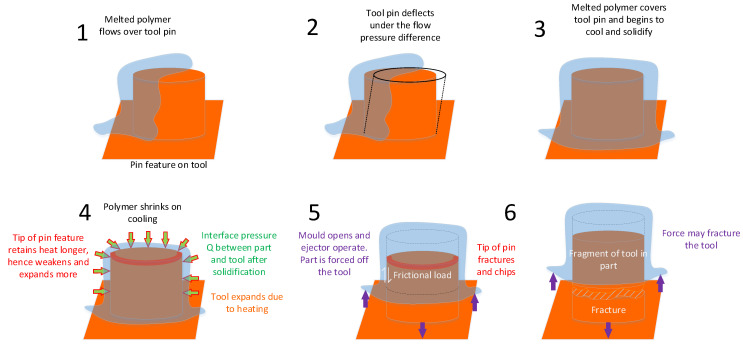
Graphical representation of different stages of injection moulding leading to failure.

We propose that during the cooling stage of the moulding process, the molten polymer cools down and shrinks (crystalising) until it is fully formed (solidified), meanwhile the 3DIM absorbs the heat from the molten polymer and expands. This shrinkage of part and expansion of the mould results in an interference pressure at the interface of the mating surface of the 3DIM tool and part. This interface pressure results in higher forces being required to eject the part out of the 3DIM during the ejection stage. Tensile stresses are developed due to frictional resistance between the 3DIM and part surface during the ejection process. These stresses, when higher than the yield strength of the 3DIM material at operating temperature, lead to 3DIM failure. Refer to stage 3 to 6 in Figure 2. The 3DIM moulds are closed during the cooling stage, and all the heat from the molten polymer is conducted into the 3DIM mould. The tip of the 3DIM features becomes hotter than the base as they have more area of the conduct the heat and this causes the tip of features to become vulnerable to failure. Stages 1 and 2 refer to the injection stage of the moulding process, which does not drastically affect the shrinkage of the polymer and thereby the failure of raised features.

### 2.2. Approach

The failure analysis of 3DIM was carried out in 2 stages, refer to Figure 1 for stage details [29]. To test the hypothesis, we examined failure samples from previous experiments, redesigned the 3DIM tools based on the learnings, re-printed the moulds and used them to injection mould until failure occurred. The moulded samples and failed 3DIM tools were analysed. A first-principles approach based on Lame’s equation for thick cylinders was used to determine the interface pressure, radial stress and hoop stress in the core pins. Finite element analysis and experimental investigations were also performed.

#### 2.2.1. Design of the Part and 3DIM Tool

**Figure 3:**
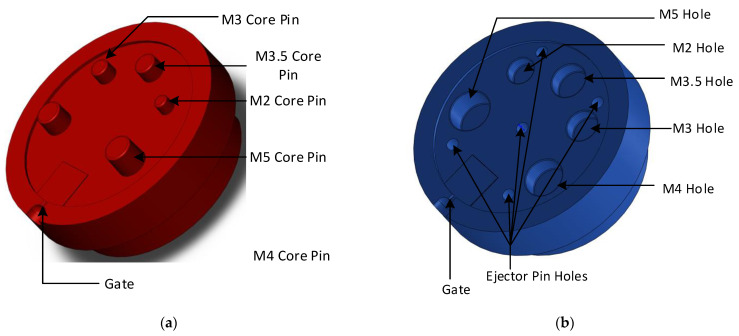
(**a**) Core side of 3DIM showing the raised features (core pins); (**b**) Cavity side of 3DIM showing the core holes and ejector pins.

Several injection moulded example parts at a local plastic injection moulding firm (Talbot Technologies Ltd., Wigram, New Zealand) were studied to determine commonly occurring features. Screw bosses were identified as a common feature among many injection moulded parts. A flat 1.5 mm thick circular plate with different size core holes was designed, the wall thickness was uniform across the whole part and a standard 1.5° draft angle was used on the raised features (an experiment with no draft angles on the moulds was also conducted for comparison purposes). The sizing for screw holes was based on the standard specifications from a threaded screw insert manufacturer SPIROL^®^. Figure 3a shows the 3DIM core and Figure 3b shows the cavity design. The pin configuration and sizing are given in Table 1. The part and tool design were carried out using SolidWorks^®^ 2019.

#### 2.2.2. Re-Design of Part and 3DIM Tool

**Figure 4:**
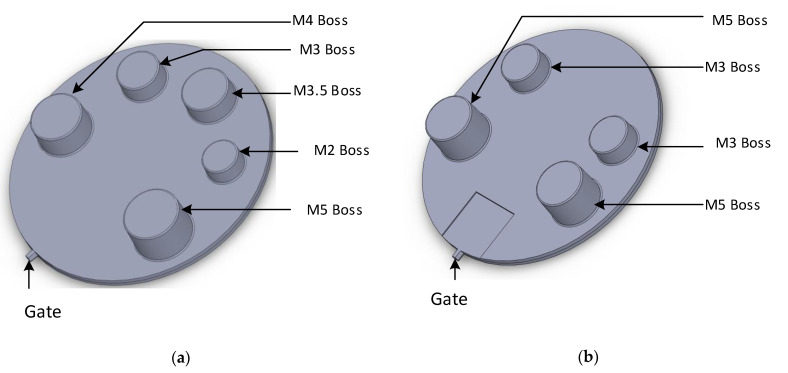
(**a**) Original part design showing 5 bosses and pin layout; (**b**) redesigned part showing 4 bosses and modified pin layout.

**Figure 5:**
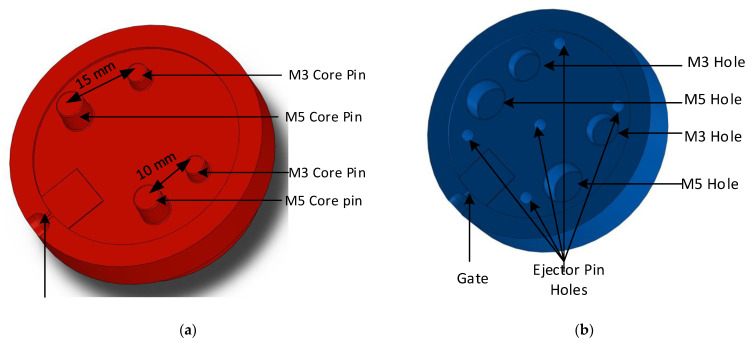
(**a**) Core side of 3DIM showing the raised features (core pins) and the distance between core pins; (**b**) Cavity side of 3DIM showing the core holes and ejector pin hole placement.

After analysing the samples from the initial moulding experiments using the 3DIM tools shown in Figure 3, we found that the M3.5 core pin was the last feature to fail in all the 3 initial experiments and the failure sequence in all 3 sets of experiments was similar. Since we wanted to confirm our hypothesis of cooling and shrinkage failure, we wanted to simplify the tool and remove the effects of differential shrinkage in the part that might be occurring due to differently sized features at different areas on the part. We eliminated 3 pin sizes and modified the tool to have two pins of different sizes at different distances from each other. The two-pin sizes (M2 and M5) used were the most commonly failed features from the previous tools. Figure 4a shows the boss layout and boss sizes on the original part and Figure 4b shows layout and sizing of the redesigned part. Since the pin layout had been modified, the 3DIM core, cavity and the MUD base also had to be redesigned. See Figure 5a for the redesigned 3DIM core insert and Figure 5b for the cavity insert.

#### 2.2.3. Theoretical Analysis

Lame’s equations for thick cylinder shrink fitting were used to calculate the resulting interference pressure between the part and the mould. The temperature conditions required were obtained via mould flow simulations using Moldex3D^®^ 2021 and validated using physical measurements via a contact thermometer HH502 from OMEGA^®^. The experimental measurements were taken at the start and end of the moulding cycle. The final part temperature difference between experimentally measured values and values from Moldex3D simulations were within 5 °C. Hence, we used the temperature data obtained from Moldex3D over the complete moulding cycle for analytical calculations and FEA simulations. Material properties for Lexan 943-A and Digital ABS were based on datasheets from suppliers [30].

#### 2.2.4. Empirical Testing

Two sets of 3DIM inserts based on the original design were printed using two different Material Jetting systems using two different resins; the details of AM systems, manufacturer, material and printing parameters used are all provided in Table 2. The same machines, material and printing parameters were then used to print two sets of the re-designed 3DIM inserts. The Projet 3500 AM system failed during empirical testing and hence only the Object Connex 350 was used for the latter stages. A design of experiments (DOE) approach was not utilised and instead, a progressive learning method was employed. After each experiment, we wanted to incorporate the learnings from the previous experiment to improve our process. The 3DIM tool cost prohibited a DOE approach.

**Figure 6:**
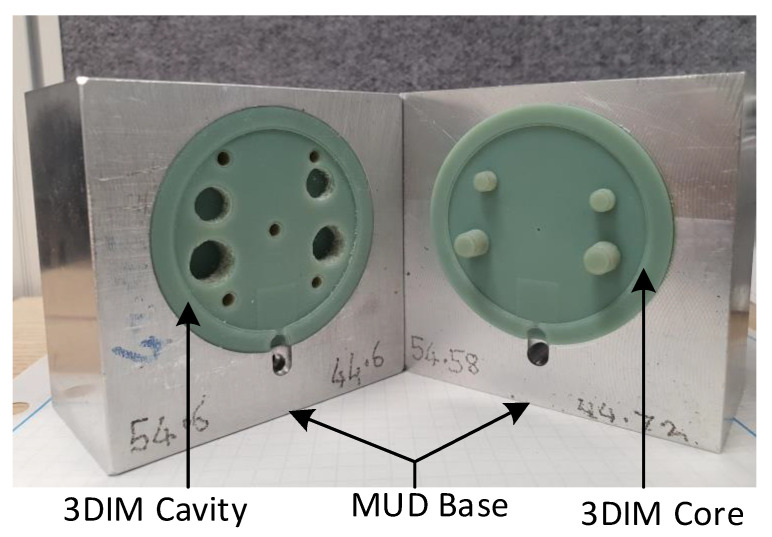
3DIM inserts (core and cavity) fitted into an Aluminium MUD base.

**Figure 7:**
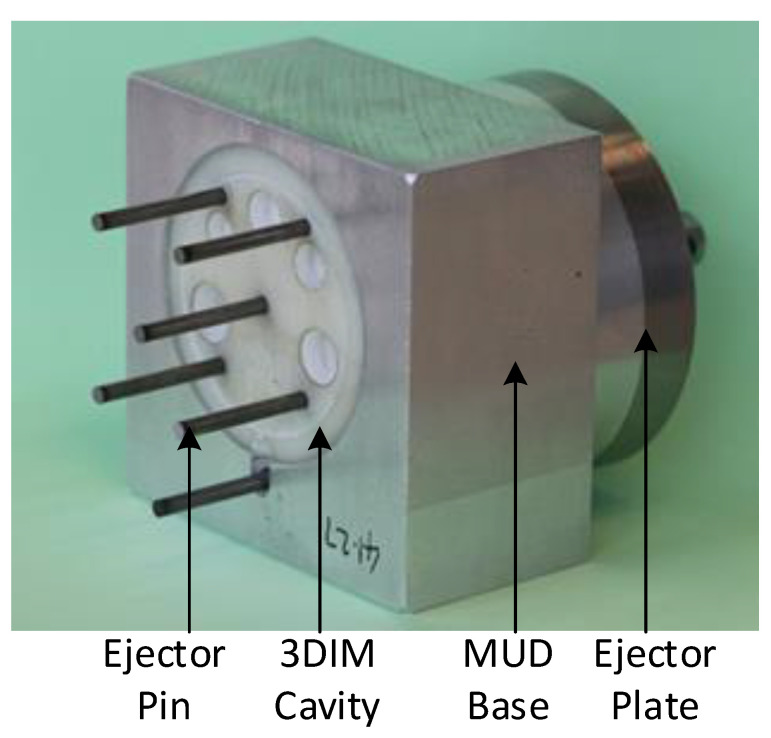
Ejector plate, pins and 3DIM insert assembled into MUD base.

A master unit die (MUD) was machined out of Aluminium 7075 and the 3DIM core and cavity inserts were fitted into the MUD base as shown in Figure 6, and a Babyplast 10/12 injection moulding machine was used for the moulding process. Figure 7 shows the ejector assembly with the ejector plates and pins. Lexan 943-A, an engineering thermoplastic from SABIC used for moulding aerospace interior parts, was used as the moulding resin. Due to the lack of hopper dryers at the University of Canterbury, we used an oven to dry the resin at 80 °C for 6 h as recommended by the material supplier to remove any traces of moisture. The material properties of the moulding resin Lexan 943-A are provided in Table 3.

The list of different pin layout, cooling time and tooling material is shown in Table 4. The process parameter setting was carried out according to the method presented in [25] and the process parameters used for the experiments are shown in Table 5. The cooling time (1), (2), (3) in Table 5 refers to the 3 different cooling times used. Cooling time here refers to the cycle time from the end of the injection stage to the mould open stage. In conventional moulding, the part is ejected immediately after the mould opens, but in our experiments, we used a small delay of 5 s to make sure the part was fully formed. To study the effect of cooling and shrinkage, all the other process parameters were kept constant between experiments and only the cooling time was adjusted.

The materials for 3DIM inserts are UV curable photopolymers (thermosets), these materials are known to have low thermal conductivity. Previously we found that the heat from the molten polymer did not penetrate to the inner layers of the inserts and was only transferring heat to a depth of about 0.25–0.4 mm [29]. For this reason, the cooling channels inside the 3DIM would need to be very close to the surface of the 3DIM for them to be effective. Drilling cooling channels close to the surface would introduce new complications for tool failure analysis and hence no cooling channels were incorporated at this stage.

## 3. Results

### 3.1. Theoretical Results

#### Interface Pressure between Part and Core

**Figure 8:**
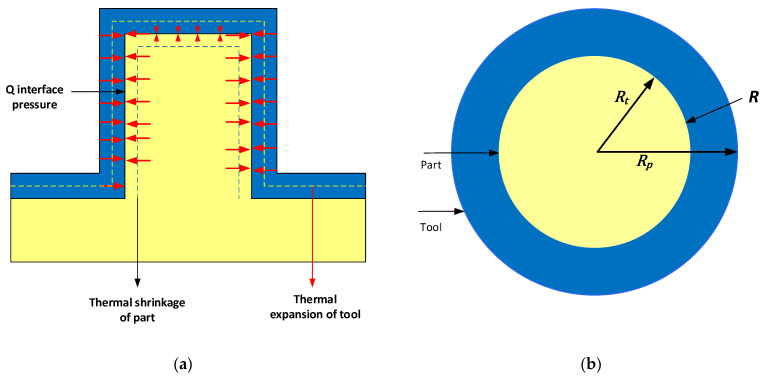
(**a**) Front section view of the raised feature showing the interface pressure due to shrinkage of tool and expansion of part.; (**b**) Top section view of the raised feature showing the interface area.

Interface pressure here refers to the pressure developed during the cooling stage of the IM process. During the cooling stage, the molten polymer inside the mould cavity is cooling and crystallising to form the part and the tool is absorbing heat from molten polymer and expanding. This shrinkage of the part and expansion of the tool results in interference at the mating surface. In Figure 8 red arrows indicate the interface pressure developed at the mating surface due to interference and the dotted lines show the potential shrinkage of part and expansion of the tool.

A detailed calculation of the interface pressure using the dimensions of the M5 core pin is presented below, Table 6 gives the description of variables and the respective values. The interface pressure is calculated using Lame’s equation for thick cylinder shrink and press fits.

The interface pressure *Q* for a shrink-fit of two different materials is given by:

(1) $$Q=[\frac{\delta }{\left[\frac{R}{E_{p}}\left\{\left(\frac{R_{p}^{2}+R_{\;}^{2}}{R_{p}^{2}-R_{\;}^{2}}\right)+\nu_{p}\right\}\right]+\left[\frac{R}{E_{t}}\left\{\left(\frac{R_{\;}^{2}+R_{t}^{2}}{R_{\;}^{2}-R_{t}^{2}}\right)-\nu_{t}\right\}\right]}]$$

The tool is solid and hence *R_t_* = 0. The interference is due to thermal effects, i.e., the shrinkage of the part (cooling) and expansion of the tool (heating) during the cooling stage of the injection moulding cycle, we have δ  = $\delta_{r}$, Thus

(2) $$Q=\left[\frac{\delta_{r}}{\left[\frac{R}{E_{p}}\left[\left\{\frac{R_{p}^{2}+R_{\;}^{2}}{R_{p}^{2}-R_{\;}^{2}}\right\}+\nu_{p}\right]+\frac{R}{E_{t}}\left[1-\nu_{t}\right]\right]}\right],$$

where,

(3) $$\delta_{r}=(\delta_{t}-\delta_{p}),$$

Since we have two different materials, and two different initial and final temperatures. The displacement for each of the material is calculated separately.

Temperature Conditions of the Part:

The part material is molten and is injected into the tool and starts to cool. Since polymers don’t have a defined melting temperature the softening point is used as the temperature at which the part starts to solidify and shrink. So $T_{ip}$ is the Vicat softening point and $T_{o}$ is, the final temperature at which the part is ejected.

(4) $$\delta_{p}=R\alpha_{p}\left(T_{op}-T_{ip}\right),$$

Temperature Conditions of the Tool:

The tool is initially at ambient temperature before the moulding cycle begins and the final temperature is the temperature measured at the end of the cycle.

(5) $$\delta_{t}=R\alpha_{t}\left(T_{ot}-T_{it}\right),$$

Since the tool is solid, the magnitude of hoop and radial stress is equal to the interface pressure.

(6) $$\sigma_{ht}=-Q,$$

(7) $$\sigma_{rt}=-Q,$$

For the part, at its inner diameter, the magnitude of radial stress is equal to the interface pressure:

(8) $$\sigma_{rp}=-Q,$$

and the hoop stress is given by:

(9) $$\sigma_{hp}=Q\left\{\frac{R_{p}^{2}+R^{2}}{R_{p}^{2}-R^{2}}\right\},$$

Substituting input values from Table 6 in Equations (1)–(9), the hoop stress, radial stress and interference pressure is obtained and are shown in Table 7.

The yield strength of the tool material (Digital ABS) at ambient temperature is reported on the datasheet as 55–60 MPa, our operating temperature for the tool ranges between 110 °C and 25 °C. The yield strength of the material at elevated temperature was previously obtained via experimental investigations [29]. The yield strength of the tool material at 100 °C is reported as 12.5 MPa and drops to 7.5 MPa at 125 °C. Comparing it to the hoop stress and radial stress on the tool we conclude that the stresses developed due to thermal loading cause the failure of core pins and the further ejection mechanism causes it to break off. However, it is important to note that this theoretical calculation is simplistic and considers just the interference of the vertical surfaces and does not incorporate the top and bottom capped surfaces. A more complex finite element analysis (FEA) is presented in the next section.

### 3.2. Finite Element Analysis

From the theoretical calculations in the previous section, we could see that the stresses developed due to thermal loading on the tool were close to the yield strength of the tool at operating temperatures. To better understand the failure mechanism, a thermo-structural FEA analysis of the 3DIM core pin assembly was performed and the results are presented below. To validate the FEA modelling and process, initially, a simple 2-part model was used to run thermo-structural analysis to obtain the interface pressure, hoop stress and radial stresses. A comparison between the theoretical and FEA values is shown in Table 8. The theoretical calculations assume there is no axial deformation, but there is slight deformation in the axial direction in the FEA model, this is the reason for the slight variation in the results.

**Figure 9:**
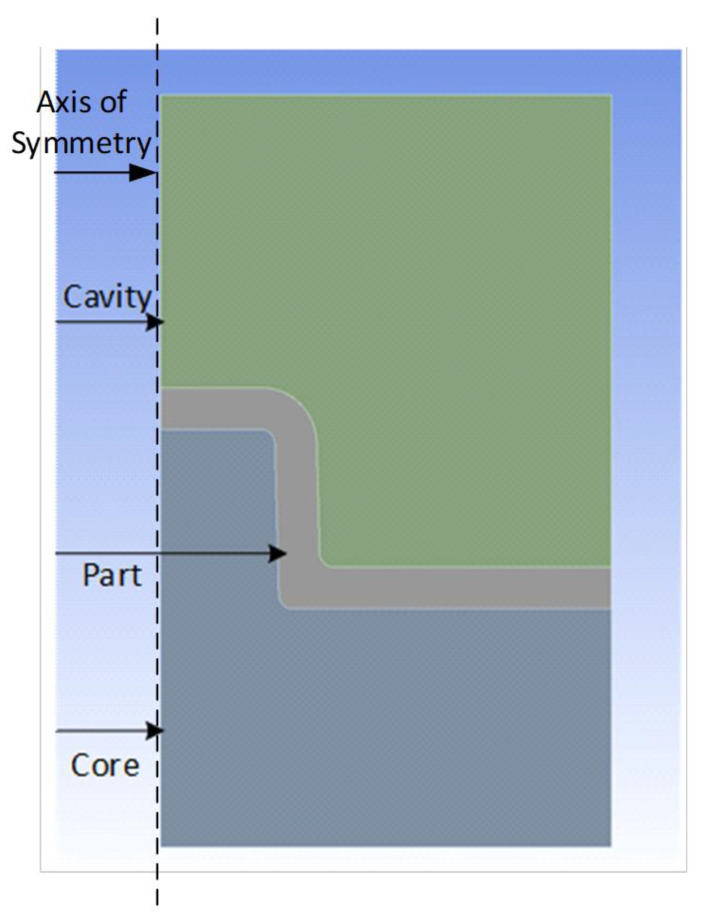
2D Axisymmetric model of the core, cavity and part assembly.

Once validated, a 2D axisymmetric model of the M5 core pin, cavity hole and the part was modelled using ANSYS^®^ 2020 and is shown in Figure 9. The FEA analysis was carried out in 2 stages: Step 1, a transient thermal analysis (cooling stage) to obtain a temperature plot and Step 2, a static structural analysis to obtain thermal stresses and interference pressure.

#### 3.2.1. Step 1: Transient Thermal Analysis

**Figure 10:**
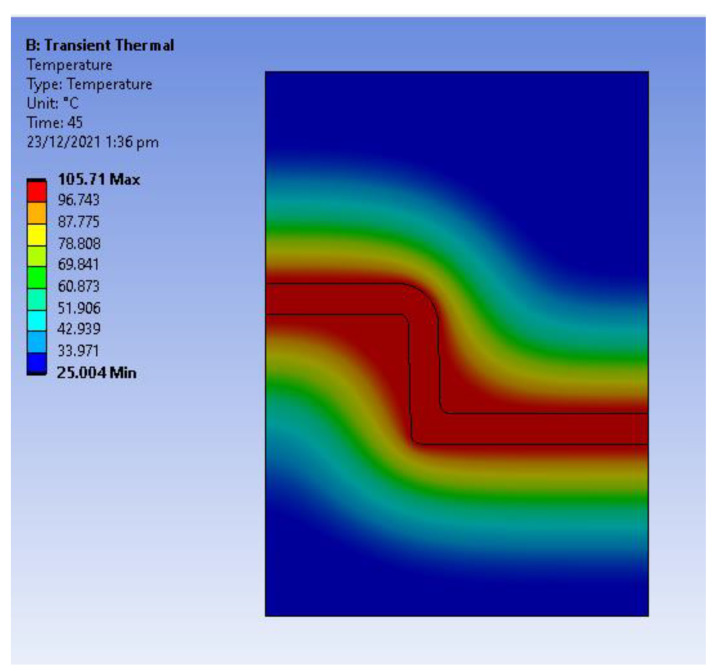
Temperature distribution at the end of the cooling stage (45 s).

The FEA analysis was carried out using ANSYS^®^ 2020. A transient thermal analysis was used to obtain the temperature distribution in the assembly at the end of the cooling stage (45 s). The input temperature required for the simulations was obtained from Moldex3D injection moulding simulations. The part temperature starting from the injection stage (molten material) to the end of the cooling stage was obtained from Moldex3D, the temperature data was also validated by measuring the part temperature at the start of the cycle (temperature of the molten material) and part temperature at the end of the cycle (fully formed part). The temperature plot for the assembly is shown in Figure 10. Quadrilateral mesh elements with 0.1 mm sizing were used. The initial part temperature is the temperature at the start of the cooling stage (217 °C) and the tool is at ambient temperature initially. The mould assembly has fixed support at the top and bottom edges and frictionless support at the right edge.

#### 3.2.2. Step 2: Static Structural Analysis

**Figure 11:**
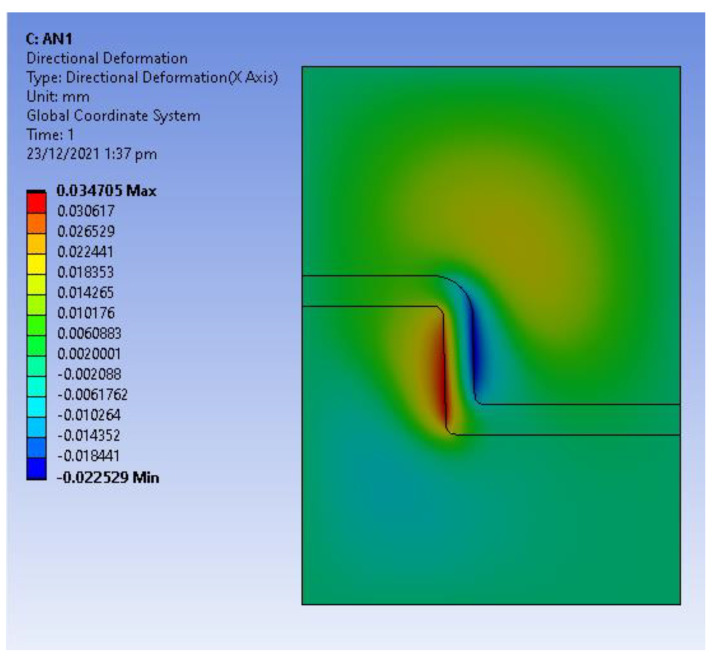
Directional deformation in *X*-axis at 45 s (expansion of tool and shrinkage of part during cooling stage).

**Figure 12:**
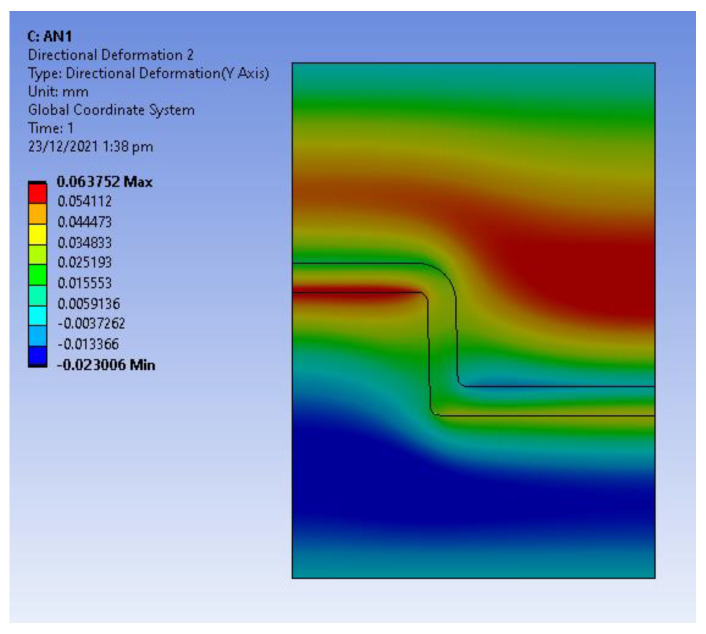
Directional deformation in *Y*-axis at 45 s (expansion of tool and shrinkage of part during cooling stage).

Static structural analysis was used to obtain the thermal stresses developed due to the expansion of the tool and shrinkage of the part as a result of the cooling stage. The temperature distribution from the transient thermal analysis was exported into a static structural analysis to determine the thermal stresses. Figure 11 shows the directional deformation in the *X*-Axis (radial deformation) and Figure 12 shows the directional deformation in *Y*-axis (axial deformation). This deformation is the reason for the high interference pressure and the hoop and radial stresses developed in the tool.

**Figure 13:**
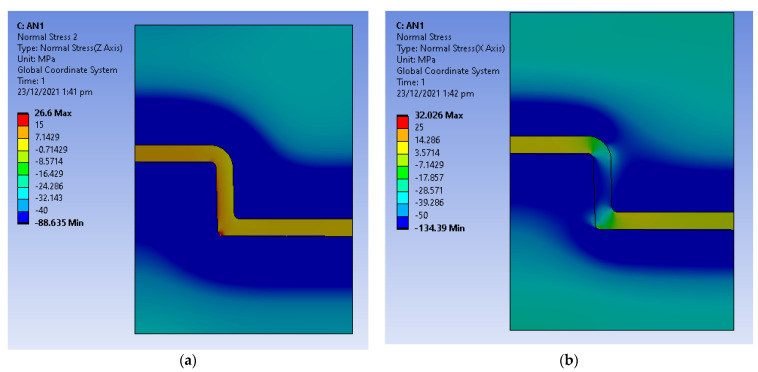
(**a**) Hoop stress at the end of the cooling stage; (**b**) Radial stress at the end of cooling stage.

The hoop stress plot on the assembly is shown in Figure 13a, and the radial stress plot is shown in Figure 13b. The tool is constrained inside an aluminium MUD base which prevents it from expanding radially outward, this puts the tool under a compressive state. The hoop and radial stress are directly dependent on the heat transfer between part and tool during the cooling stage.

#### 3.2.3. Step 3: Static Structural Analysis

In our case, the core side of the 3DIM tool is attached to the fixed half of the moulding machine and the cavity side is attached to the moving half of the moulding machine. Once the cooling stage is completed, the moving half is pulled back and the part stays attached to the cavity. To simulate this in FEA, we used a displacement boundary condition on the core side of the tool (−6 mm in Y-axis) and the cavity side of the tool was fixed. This was carried out in order to simplify the number of constraints and steps. A 0.2 co-efficient of friction was assumed between the part and moving half of the mould (core) and bonded contact was assumed at the part and non-moving half of the mould (cavity). This is a simplified assumption, in our experiments, the part was stuck to the cavity in the majority of cases (95% of the time) and to simulate this a bonded contact assumption was used. Since this is a simplified 2D axisymmetric model of one core pin, without the use of bonded contact, the part was being stuck to the core instead of the cavity. We suspect that due to simplification of the geometry, the differential shrinkage of the part is not considered and this shrinkage might be one of the reasons the part during experiments is stuck to the cavity and not the core. This is an exploratory study of the effects of cooling on the raised features.

**Figure 14:**
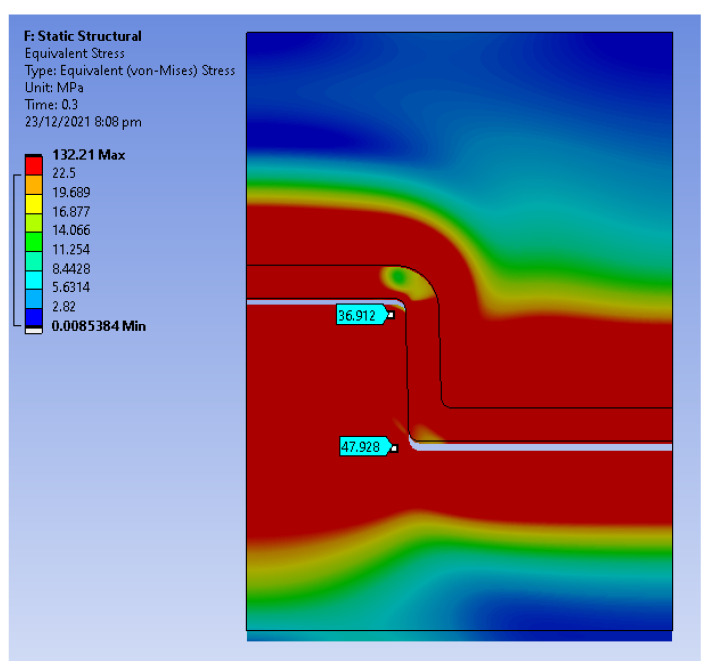
Equivalent stress distribution at 0.1 s.

**Figure 15:**
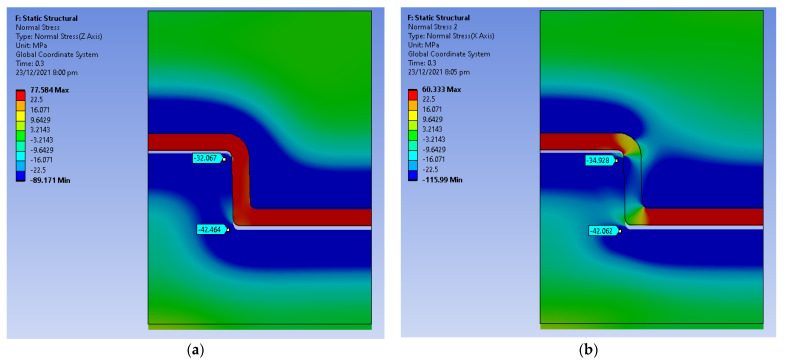
(**a**) Hoop Stress at 0.3 s (0.5 mm displacement); (**b**) Radial Stress at 0.3 s (0.5 mm displacement).

**Figure 16:**
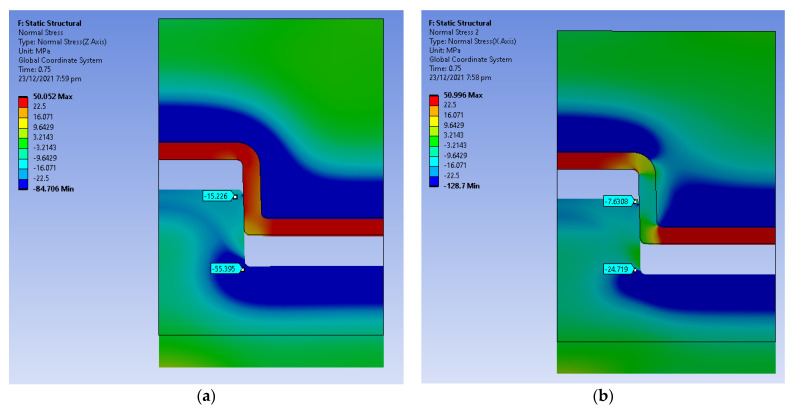
(**a**) Hoop Stress at 0.75 s (3 mm displacement); (**b**) Radial Stress at 0.75 s (3 mm displacement).

The displacement was simulated over a period of 1.5 s. Figure 14 shows the equivalent stress distribution at a time step of 0.1 s where the core pin has displaced 0.3 mm. The equivalent stress at the bottom and the top edge is higher than the yield strength of the material. Figure 15a shows the hoop stress distribution and Figure 15b shows the radial stress distribution at a time step of 0.1 s. There is a sudden increase in hoop and radial stress from the end of the cooling stage to the first displacement. We suspect that the high radial and hoop stress on the tool results in chipping of the part when it is ejecting, this is consistent with the experimental results. Figure 16a,b show the radial and hoop stress on the tool at a time step of 0.75 s, as the tool slides, the contact area reduces and the stresses reduce.

High compressive values of hoop stress are evident in the tool (Figure 16a), and this is attributed to the tool being constrained on its outer surface (right edge of the model). The constraint represents the situation of the tool being inserted into a MUD base, which adds a constraint against expansion. The simulation was also run without this boundary condition, and the results (not shown) indicated lower hoop stress as it allowed free expansion of the tool.

### 3.3. Experimental Results

#### 3.3.1. Cooling Time 1: 45 s Cooling Time

**Figure 17:**
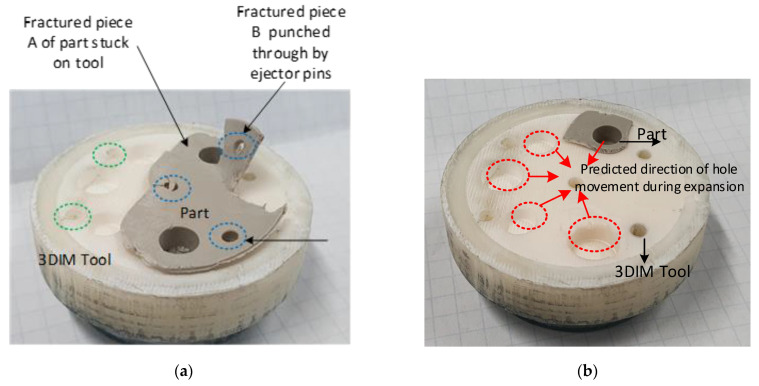
(**a**) Broken part with punched ejector holes stuck inside 3DIM cavity; (**b**) 3DIM cavity with broken part stuck inside after ejection and highlighting the potential expanded state of the cavity holes in a red circle.

3DIM inserts for this experiment were printed using the material Visijet M3-X. The process parameter shown in Table 4 was used with a 45 s cooling time. After each moulding cycle, the moulds were kept open and compressed air cooling was used in between cycles to cool the 3DIM inserts back to 28 °C. This was the only experiment in which the 3DIM cavity inserts were observed to be deteriorating. In this case, the 3DIM inserts were seen to be deteriorating from the 3rd moulding cycle. The first moulding cycle was normal and the part ejected as intended, but during the subsequent moulding cycle, the part became stuck inside the cavity side of the tool and required manual ejection (pulling the part using pliers). On the 4th moulding cycle during the part ejection, the part was broken. Figure 17a shows the broken part and the tool, the part was firmly stuck inside the tool and the ejector pins punched a hole through the part during the ejection stage. We concluded that the cavity failure was due to a combination of insufficient draft angle and excessive shrinkage of the part. Figure 17b shows the state of the tool after ejection. Since the 3DIM is enclosed inside an aluminium MUD base, the cavity holes become smaller when the 3DIM expands during the cooling stage. We believe that this results in higher frictional forces and results in edge chipping of the 3DIM cavity.

**Figure 18:**
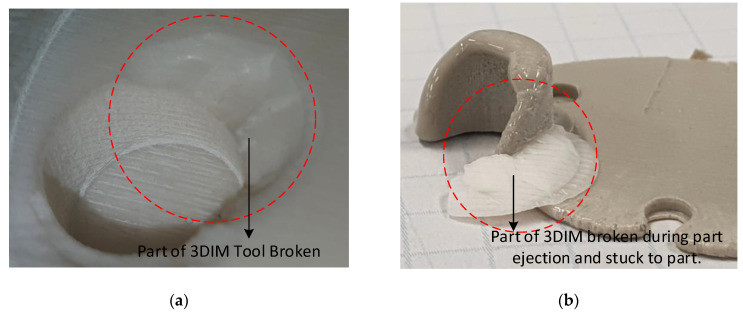
(**a**) 3DIM insert showing part missing from the tool that was broken during ejection; (**b**) Broken part of 3DIM tool material stuck to the moulded part. (White material is the 3DIM tool and grey material is the part).

The edges on the 3DIM cavity holes were chipping during ejection and becoming stuck onto the part. Figure 18a shows the broken edge of the 3DIM cavity and Figure 18b shows the broken edge piece of the tool stuck onto the part. The edges of the cavity holes on the side closer to the central location of the tools were seen to be deteriorating. We suspect that these failures are not solely due to interference pressure, but due to inadequate draft angle which resulted in aggravating the effects of interference pressure and ejection. The 3DIM inserts were printed again with an increased draft angle of 1.5 degrees and the tool life increased from 3 shots to 7 shots before any sign of deterioration was observed. These failures can be compared to the FEA stress states shown in Figure 14 and Figure 15.

##### Core Failures

Failure of the core insert was first observed on the 3rd moulding cycle. Analysing the moulded part from the third shot, we could identify the raised feature (core pin) of the 3DIM tool that had fractured at the base and stuck onto the part during the ejection stage.

**Figure 19:**
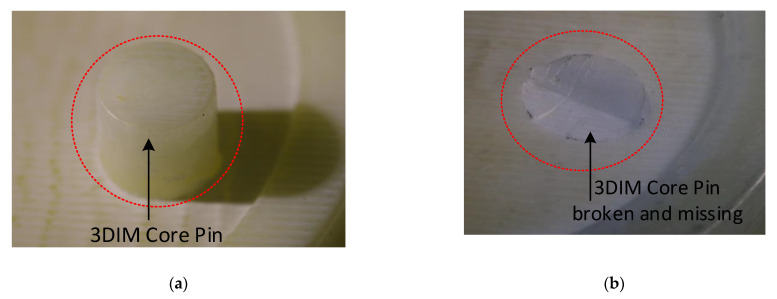
(**a**) 3DIM tool with an intact core pin before moulding; (**b**) 3DIM tool with a broken a missing core pin after moulding and part ejection during 3rd shot.

**Figure 20:**
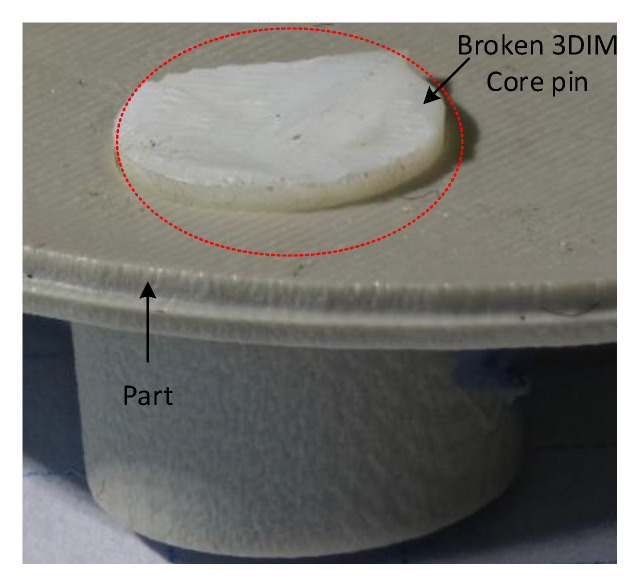
M5 core pin on the 3DIM tool broken and stuck inside the part during ejection. (White coloured material is the tool and grey coloured material is the part).

In Figure 19a the M5 core pin before the moulding cycle began is shown, subsequently in Figure 19b the M5 core pin after the 3rd moulding cycle is shown. The M5 core pin is no longer present here and was fractured during the moulding cycle and stuck inside the part during ejection. In Figure 20, the 3rd part during which the M5 core pin broke off can be seen stuck inside the part. The core pin is broken at the base but is protruding outside, this is because the part starts to eject and the frictional resistance increases and breaks the pin, before breaking some portion of the part is ejected and that is why the pin protrudes. This can also be seen in Figure 15, the core starts moving away and the part is slightly ejected, but the stresses resulted in failure of the pin.

We attributed these raised feature (core pin) failures to interference pressure and ejection forces. The cooling rate is an important factor that affects part shrinkage and in the case of a 3DIM tool, it also affects the tool expansion. In this case, the high shrinkage is due to the slow rate of cooling. The longer cooling time also means the 3DIM tool is heating up and expanding which results in a high interference at the part-tool mating surface. The interference pressure along with the rough surface due to the layered printing process results in a higher frictional force during ejection which causes the 3DIM raised features to break off. The higher the interference pressure, the higher the ejection forces, which effectively means longer cooling cycles (slower cooling rate) lead to abrupt failure of the 3DIM tool. Referring to the stresses in Figure 16a, this high stress at the base of the pin leads to the raised feature failure.

#### 3.3.2. Cooling Time 2: 30 s Cooling Time

3DIM Inserts for this experiment were printed using Visijet M3-X and Digital ABS. The process parameter (2) shown in the table was used with a 30 s cooling time. In this set of experiments the mould was initially kept closed for 30 s after injection and the parts were ejected immediately after mould opening.

##### Cavity Failures

**Figure 21:**
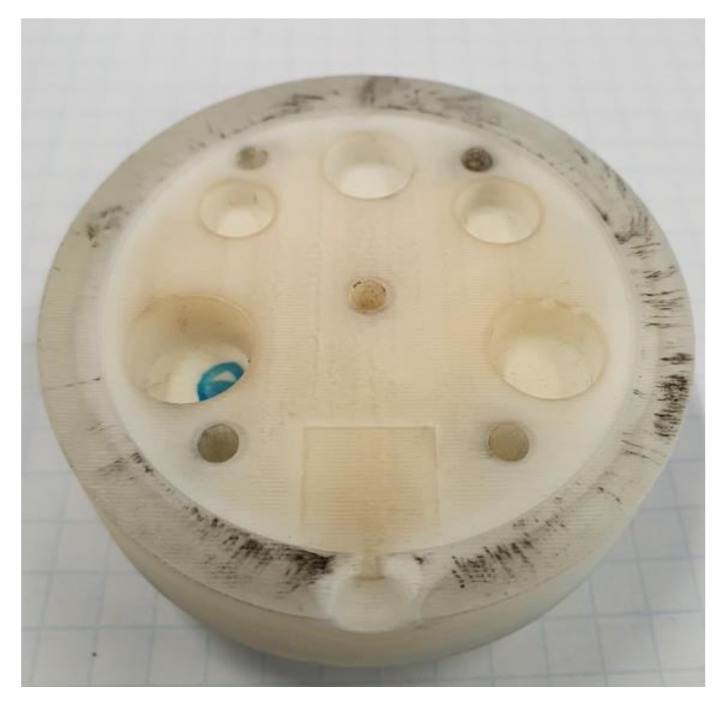
3DIM cavity showing no signs of deterioration after 10 moulding shots.

There were no observed cavity failures during this experiment, Figure 21 shows the 3DIM cavity after 10 Shots.

##### Core Failures

In this experiment we reduced the cooling time to confirm our hypothesis that reducing cooling time would decrease the interference pressure and thereby reduce the frictional resistance during the ejection stage leading to a higher 3DIM tool life.

**Figure 22:**
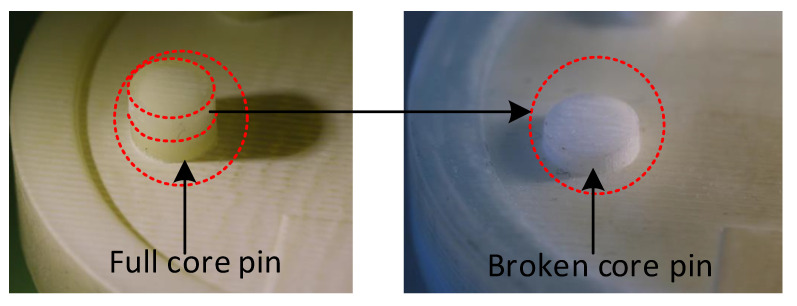
M5 core pin on the 3DIM tool printed using Visijet M3-X before moulding and after initial chipping failure on the 5th shot.

To reduce the interference, we needed to reduce thermal shrinkage of the part and thermal expansion of the tool. This was achieved by changing the cooling regime. Opening the mould after 30 s of closed cooling, meant the part was exposed to ambient air, which leads to the part cooling down faster than when it was kept closed for 45 s. Opening the mould also meant that the tool would stop heating up as it was exposed to ambient air. This setting should result in comparatively lower interference than in experiment 1. In theory the complete moulding cycle time for both experiment 1 and 2 is 45.15 s, but in experiment two, interference was lesser because the cooling rate was faster as it was exposed to ambient air after 30 s. In experiment 1 the M5 core pin fractured completely and broke on the 3rd moulding cycle and in experiment 2 with reduced cooling time the M5 core pin started chipping on the 5th moulding cycle, see Figure 22. The other core pins (raised features) were also seen to be deteriorating progressively.

While reducing the cooling time reduced the interference and frictional forces during ejection and thereby the probability of abrupt failures, the 3DIM raised features were still chipping off and becoming stuck inside the part during ejection.

**Figure 23:**
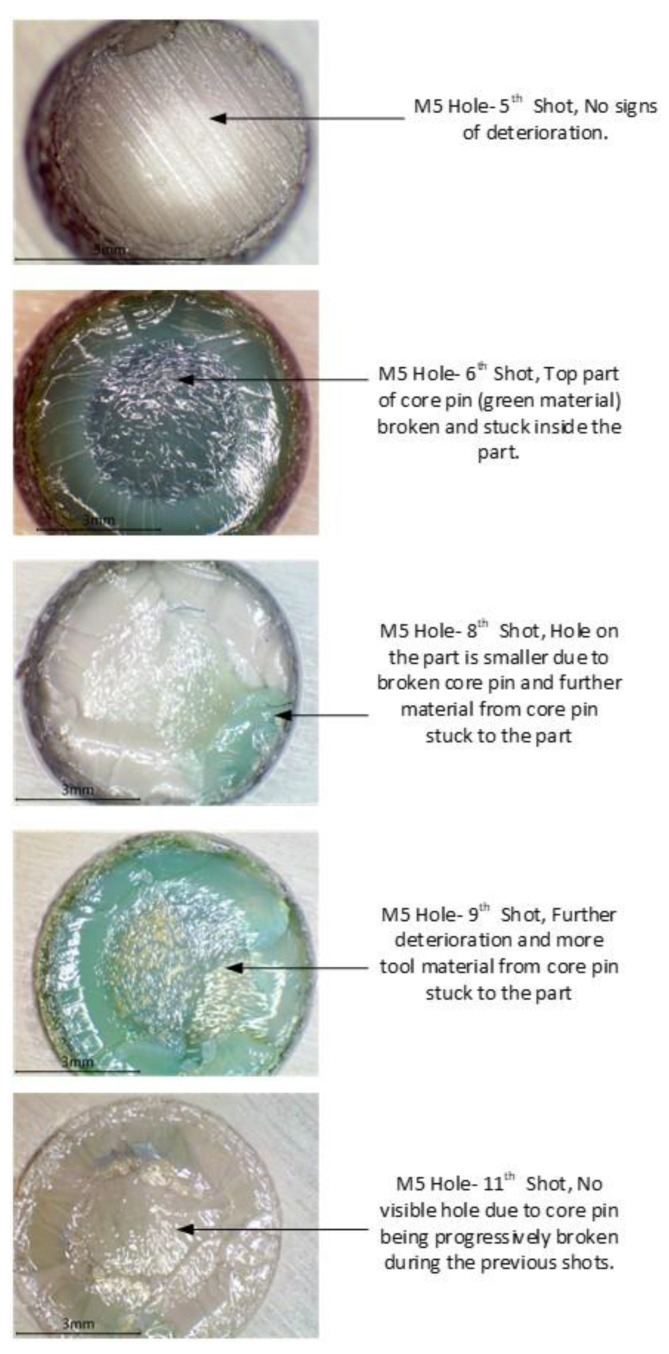
Progressive reduction in depth of the M5 core hole as a result of deterioration of the M5 core pin. (The green material is the core pin that been chipped and stuck onto the part during ejection).

In Figure 23 the progressive deterioration of the raised feature can be clearly seen. Comparing the failure of the M5 core pin we could see that reducing the cooling time led to reduced frictional resistance and thereby transitioned from abrupt failure to progressive deterioration. An oblique view of the failure and progressive deterioration of the core pin has previously been documented [29].

All the above experiments were performed using the 5-pin layout 3DIM tool design shown in Figure 3. Since the hypothesis involved shrinkage of the part, we wanted to rule out the possibility of the pin configuration causing differential shrinkage around the part area. The experiments were also repeated with the modified 4 pin layout 3DIM tool design shown in Figure 5. After examining the moulded parts, we concluded that the failure patterns observed were similar to the previously observed experiment.

#### 3.3.3. Cooling Time 3: 15 s Cooling Time, Increased to 20 s

3DIM inserts for this experiment were printed using Digital ABS and the printing parameters shown in Table 2. Based on the results from previous experiments, we wanted to further reduce the cooling time to determine if failure of raised feature was avoidable.

**Figure 24:**
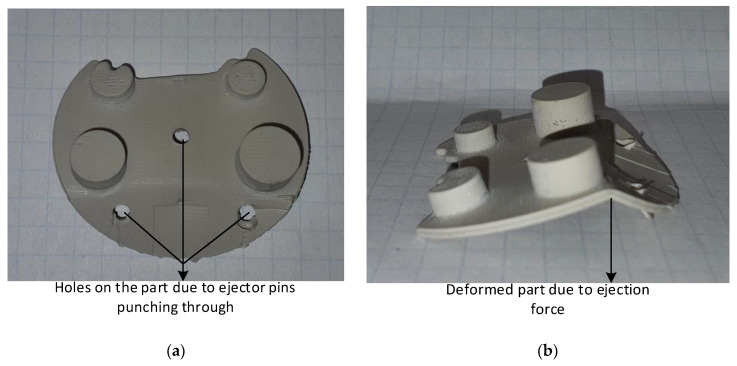
(**a**) Part showing holes punched by ejector pins as the parts were not completely solidified; (**b**) Deformed part during ejection stage as the part was not completely solidified.

The 15 s cooling time was used to try and further reduce the interference thereby reducing the ejection forces. This experiment was only run for 5 moulding cycles, because the cooling time was in-adequate and the parts were not fully formed (solidified) before ejection. The data sheet from moulding material suppliers recommend parts to be ejected at 80 °C, the part temperature when measured after ejection was averaging 105 °C. The external skin layer of the part was solidified, but the internal layers were still in the process of solidifying. When the ejector pins operated, instead of ejecting the part, they punched holes on the part as shown in Figure 24a,b, the parts also deformed due to the ejection force as they had not yet solidified when the ejector pins operated.

**Figure 25:**
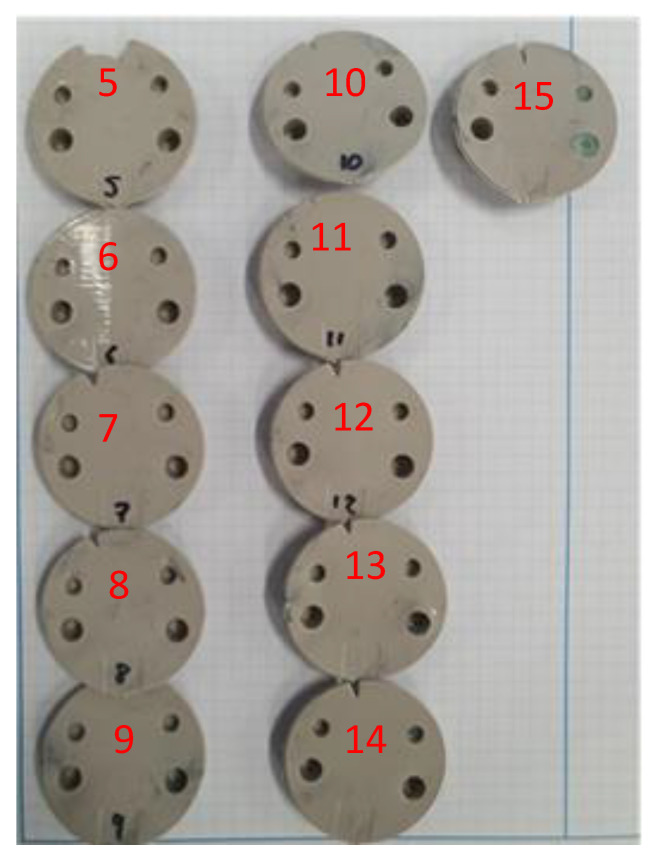
Part sequence, moulded using 20 s cooling time. Numbers refer to shot. Parts 1–4 not shown as they are were not fully formed due to shorter cooling time. Mould failure occurred at shot 14.

While lowering the cooling time is an appropriate method to decrease interference pressure, the cooling time also has to be sufficient to enable the parts to fully form and solidify before they can be ejected. Since the tool had not shown any signs of deterioration, we decided to continue the experiment and increased the cooling time to 20 s. Figure 25 shows the sequence of parts moulded using the 20 s cooling time. The first sign of failure on the M5 core pin was seen after 14 shots. This was a significant increase from the previous 7th shot failure.

## 4. Discussion and Conclusions

### 4.1. Findings

The rate of cooling or cooling time is one of the key factors that affect part shrinkage. In conventional moulds, cooling only affects part shrinkage and thereby the part quality and generally has no adverse effect on the tool life. Whereas in 3DIM tools, cooling affects both the part quality and tool life. Cooling time was found to be a critical factor in 3DIM tool life; abrupt failure of raised features on 3DIM tools could be avoided by using a suitable cooling regime. Longer cooling time tends to cause higher part shrinkage and 3DIM tool expansion which led to high interference pressure and thereby caused abrupt failures of raised features during ejection. When the cooling times were reduced, we could see a progressive deterioration of the 3DIM tool rather than abrupt failures. In the majority of use cases, the tool showing signs of progressive deterioration was still usable but the tool with abrupt failures could not be used due to safety issues. The current work has examined the shrinkage and resulting inference stresses for raised parts, specifically circular cross-section bosses. The principles are expected to generalise to other raised features such as ribs and thin walls. For flat parts in the plane of the parting line, the results are not expected to be relevant, as these features are not subject to appreciable frictional resistance at ejection.

From results in experiments 1, 2 and 3, we could see the tool life increase with decreasing cooling time. In Experiment 1 with a 45 s cooling time, we had an abrupt failure on the 3rd shot. Whereas, in Experiment 2 with a 30 s cooling time, we had progressive deterioration of the core pin starting on shot 7 and failing completely on the 9th shot. Further reducing the cooling time to 20 s led to a 3DIM tool showing no signs of deterioration until the 14th shot. This was the lowest cooling time at which a part could be ejected, and lower cooling times lead to unformed parts. While we believe cooling channels would not help in reducing the cooling times, due to the low thermal conductivity of polymers, we believe a different approach, such as compressed air cooling, could decrease the interference

### 4.2. Limitations of the Research

The rate of cooling is also a critical factor for part quality, in this research we did not consider the part quality and thereby the effects of cooling rate/time on part quality is not discussed. Flash cooling will result in brittle parts with poor mechanical properties that might not be suited to their application. The compressed air cooling was carried out manually between each injection moulding cycle and the tool temperature was measured at 3 different locations before the next moulding cycle. There is a possibility that the tool was not uniformly cooled and there might have been some hot spots that lead to further worsening of interference pressure

### 4.3. Implications for Future Research

The cooling stage of the moulding cycle is the longest (45 s), this is a significant amount of time for heat transfer between the molten polymer and 3DIM tool. The thermal stresses developed are a critical factor for tool failure. In conventional moulding the cooling stage only affects the part quality and cycle time, therefore the effect of cooling on tool life has not been of much interest. It is important for tool designers to consider the thermal stresses on raised features of the 3DIM and equally important for technicians to use an appropriate cooling time. The conventional tool design principles of 0.5-degree draft angles, heated moulds for easier flow of material are some of the common pitfalls when using 3DIM tools.

### 4.4. Conclusions

This paper has evaluated the hypothesis and confirms that a raised feature on a 3DIM tool experiences a compressive stress, that is developed due to the interference between part and tool during the cooling and tensile stresses that is developed due to the frictional resistance between the part and the tool during the ejection stage.

The interference was found to be increasing with increasing cooling times and thereby also increasing the frictional resistance during the ejection stage. The hypothesis was evaluated on theoretical and experimental grounds.

## Figures and Tables

**Table 1:** Dimensions of the core pin and distance from the gate (extracted and reproduced from Bagalkot et al. (2017) with permission [29]).

Name	Diameter (mm)	Height (mm)	Aspect Ratio	Distance from Gate (mm)
M2 Core Pin	3.63	3.18	1.14	35.8
M3 Core Pin	4.75	3.56	1.33	35.8
M3.5 Core Pin	5.54	3.81	1.45	39.45
M4 Core Pin	6.38	4.7	1.35	23.71
M5 Core Pin	7.16	6.35	1.12	23.71

**Table 2:** Material specification and printing parameters used for printing 3DIM inserts gate (extracted and reproduced from Bagalkot et al. (2017) with permission [29]).

	MJ Machine 1	MJ Machine 2
Machine	Projet 3500	Object Connex 350
Manufacturer	3D Systems	Stratasys
Material	Visijet M3X	Digital ABS
Layer Thickness	30 Microns	30 Microns
Print Mode	Not Applicable	Matte
Cleaning	Water Jet Cleaning	Water Jet Cleaning

**Table 3:** Material Properties of Lexan 943-A.

Description	Value
Density	1.2 g/cm^3^
Melt Flow Rate	9.00 cm^3^/10 min
Drying Temperature	120 °C
Max Moisture	0.020%
Hopper Temperature	60 °C
Melt Temperature	280–300 °C
Mould Temperature	80–100 °C

**Table 4:** List of cooling times, tooling material and pin layout used.

Cooling Time	Material	Pin Layout
45 s	Digital ABS	5 Pin
45 s	Visijet M3-X	5 Pin
30 s	Digital ABS	5 Pin
30 s	Visijet M3-X	5 Pin
30 s	Digital ABS	4 Pin
15 s	Digital ABS	4 Pin

**Table 5:** Injection moulding process parameters.

Description	Value
Resin	Lexan 943-A
Type	Polycarbonate
Mould Temperature	28 °C
Melt Temperature	300 °C
Injection Pressure	60 MPa
Fill Time	0.2 s
Cooling Time (1)	45 s
Cooling Time (2)	30 s
Cooling Time (3)	15 s
Mould Open Time	Open until the mould temperature returned to 28 °C

**Table 6:** Description of the input variables and values for M5 core pin.

Symbol	Description	Value	Units
*R*	Interface Radius	3.58	mm
*R_p_*	Part Radius (Outer)	5.08	mm
*R_t_*	Tool Radius (Inner)	0	mm
$\nu_{p}$	Poisson’s Ratio-Part material	0.41	
$\nu_{t}$	Poisson’s Ratio-Tool material	0.36	
*E_p_*	Youngs Modulus-Part	2350	MPa
*E_t_*	Youngs Modulus-Tool	2600	MPa
*α_p_*	Co-efficient of thermal expansion (Part)	7.00 × 10^−5^	1/°C
*α_t_*	Co-efficient of thermal expansion (Tool)	1.50 × 10^−4^	1/°C
*T_ip_*	Initial part temperature	120	°C
*T_op_*	Final part temperature	90	°C
*T_it_*	Initial tool temperature	25	°C
*T_ot_*	Final tool temperature	90	°C

**Table 7:** Descriptions of output variables and values for M5 core pin.

Symbol	Description	Value	Units
*Q*	Interface Pressure	8.39	MPa
*T_ip_*	Initial part temperature	120	°C
*T_op_*	Final part temperature	90	°C
*T_it_*	Initial tool temperature	25	°C
*T_ot_*	Final tool temperature	90	°C
*σ* *_hp_*	Hoop Stress on part at *R_p_*	24.95	MPa
*σ* *_ht_*	Hoop Stress on tool at *R_t_*	−8.39	MPa
*σ_rp_*	Radial Stress on part at *R_p_*	−8.39	MPa
*σ_rt_*	Radial Stress on tool at *R_t_*	−8.39	MPa

**Table 8:** Comparison of theoretical vs FEA results carried out for validation of the FEA modelling process.

Description	FEA	Theoretical	Units
Interface pressure	8.59	8.39	MPa
Hoop stress part	26.68	24.95	MPa
Hoop stress tool	−8.59	−8.39	MPa
Radial stress part	−8.59	−8.39	MPa
Radial stress tool	−8.59	−8.39	MPa

## Data Availability

Not applicable.
